# 169 Strategies for recruitment of adolescent girls into physical activity programmes: a systematic review

**DOI:** 10.1093/eurpub/ckae114.109

**Published:** 2024-09-26

**Authors:** Tanya O’Brien, Catherine Darker, David Mockler, Emer Barrett

**Affiliations:** Department of Physiotherapy, The University of Dublin, Trinity College Dublin, Dublin, Ireland; Discipline of Public Health and Primary Care, The University of Dublin, Trinity College Dublin, Dublin, Ireland; School of Medicine, The University of Dublin, Trinity College Dublin, Dublin, Ireland; Department of Physiotherapy, The University of Dublin, Trinity College Dublin, Dublin, Ireland

## Abstract

**Purpose:**

Physical activity is essential for youth physical and mental health, yet just 15% of adolescent girls worldwide meet the World Health Organization guideline of 60 minutes of moderate-to-vigorous physical activity per day. A persistent challenge faced by physical activity providers, however, is recruiting adolescent girls into their programmes. This review aims to provide quantitative evidence-based recommendations for optimal recruitment practices for this cohort.

**Methods:**

Five electronic databases were searched (Embase, Medline, CINAHL, Web of Science, and Cochrane Library – Central Trial Registry) including database-specific terms, truncations, and synonyms combining ‘adolescent females’, ‘recruitment’, and ‘physical activity’ to identify randomised controlled trials of physical activity interventions for adolescent girls worldwide. Hand searches of references were also conducted. Data was extracted regarding study, participant, and intervention characteristics, pre-determined recruitment goals, recruitment strategies employed, and the number of participants screened, eligible, approached, randomised, and retained. Outcomes included whether recruitment goals were met, recruitment rate, and any revised recruitment measures implemented mid-study.

**Results:**

After removal of duplicates (n = 71), 5460 articles were screened by title and abstract independently by two reviewers. 70 full-text articles were screened by two reviewers, resulting in 26 included studies. Preliminary analysis showed that most physical activity programmes were school-based (76.4%), multicomponent (65.4%), and designed based on behaviour change theories (80.8%). All studies employed multiple recruitment strategies such as distributing information to eligible students in schools (14 studies), researchers presenting directly to eligible students at their school (10), teachers assisting with recruitment (6), providing small gifts, vouchers, or the ability to earn awards for participation (5 each), and posting flyers in schools (4). The mean recruitment rate for adolescent girls into physical activity randomised controlled trials was 60.4% (SD = 26.94), with high variability (23.6%-99.2%). Ongoing analysis will provide regression results regarding the effectiveness of recruitment strategies employed and their impact on recruitment outcomes.

**Conclusions:**

Application of the findings of this review by physical activity programme providers may enhance their recruitment processes, potentially increasing teenage girls’ physical activity enrolment worldwide.

**Funding:**

This research was funded by the Irish Research Council and the Dublin City Sport & Wellbeing Partnership through the Enterprise Partnership Scheme.

